# The Tremont and Revere Houses in Boston

**Published:** 1874-12

**Authors:** 


The Tremont and Revere Houses in Boston.—Travellers who have stopped at either of these first class hotels will agree with us that there are few better ones in the United States. The proprietors, Messrs. Chapin, Gurney & Co., are practical and experienced hotel managers, and seem to possess the remarkable faculty of making all guests contented and happy. These hotels are delightfully situated near Boston Common, and not far from the prominent places of amusement.

